# Bacterial and Viral Infection and Sepsis in Kidney Transplanted Patients

**DOI:** 10.3390/biomedicines10030701

**Published:** 2022-03-18

**Authors:** Alberto Mella, Filippo Mariano, Caterina Dolla, Ester Gallo, Ana Maria Manzione, Maria Cristina Di Vico, Rossana Cavallo, Francesco Giuseppe De Rosa, Cristina Costa, Luigi Biancone

**Affiliations:** 1 Renal Transplant Center “A. Vercellone”, Nephrology, Dialysis, and Renal Transplant Division, “Città Della Salute e Della Scienza” Hospital, Department of Medical Sciences, University of Turin, 10126 Turin, Italy; alberto.mella@unito.it (A.M.); filippo.mariano@unito.it (F.M.); cdolla@cittadellasalute.to.it (C.D.); egallo2@cittadellasalute.to.it (E.G.); amanzione2@cittadellasalute.to.it (A.M.M.); mdivico@cittadellasalute.to.it (M.C.D.V.); 2 Microbiology and Virology Unit, University of Turin, 10126 Turin, Italy; rossana.cavallo@unito.it (R.C.); cristina.costa@unito.it (C.C.); 3 Department of Medical Sciences, Infectious Diseases, University of Turin, A.O.U. Città Della Salute e Della Scienza di Torino, 10126 Turin, Italy; francescogiuseppe.derosa@unito.it

**Keywords:** kidney transplantation, viral infection, bacterial infection, sepsis

## Abstract

Kidney transplanted patients are a unique population with intrinsic susceptibility to viral and bacterial infections, mainly (but not exclusively) due to continuous immunosuppression. In this setting, infectious episodes remain among the most important causes of death, with different risks according to the degree of immunosuppression, time after transplantation, type of infection, and patient conditions. Prevention, early diagnosis, and appropriate therapy are the goals of infective management, taking into account that some specific characteristics of transplanted patients may cause a delay (the absence of fever or inflammatory symptoms, the negativity of serological tests commonly adopted for the general population, or the atypical anatomical presentation depending on the surgical site and graft implantation). This review considers the recent available findings of the most common viral and bacterial infection in kidney transplanted patients and explores risk factors and outcomes in septic evolution.

## 1. Introduction

Kidney transplanted patients (KTRs) are a unique population with intrinsic susceptibility to viral and bacterial infections, mainly due to continuous immunosuppression [1,2]. Infectious episodes remain one of the most important causes of death in this group [3,4], with different risks according to the degree of immunosuppression, time after transplantation, type of infection, and patient conditions. The persistence of an immunosuppressive state may lead to both viral and bacterial infections from atypical and opportunistic agents, posing some questions about therapeutic management [1]. This review considers the recent available findings of the most common viral and bacterial infection in kidney transplanted patients and explores risk factors and outcomes in septic evolution.

Considering the specific, atypical, and mutating characteristics of the pandemic SARS-CoV2, which need extensive and separate discussion, this infection is not mentioned in this review.

## 2. General Considerations about Infection in Kidney Transplanted Patients

Cornerstones of transplant infectious disease management in KTRs are: be rapid, be specific, but also be cautious (e.g., in reducing immunosuppressive drugs) (Table 1).

Timing of diagnosis and appropriate therapy are a crucial part of infective management in this frail population. Generally, in the timeline of common infectious episodes, the first month’s diseases are pre-existent, nosocomially-acquired (including wound and surgical-site infections) or, in low percentage, donor-derived; after this period and during the first year (especially between one and six months), the “pressure” of high immunosuppression exposes the patient to opportunistic infections [1]. However, some specific characteristics of transplanted patients may cause a diagnostic delay, such as the absence of fever or inflammatory symptoms due to immunosuppressive therapy, the negativity of serological tests commonly adopted for general characteristics, or the atypical anatomical presentation depending on the surgical site and graft implantation. By the way, the diagnosis should be promptly run out with the combination of in-depth radiological (i.e., CT and MRI) and microbiological analyses, while also considering invasive tests to avoid unnecessary or prolonged antibiotic/antiviral therapies, especially considering the risk of toxic reactions and the emergence of resistant strains [1,5].

Every drug has a specific effect on the immune system, and assessing the effective net state of the immunosuppression is difficult but crucial to improve the outcome (as discussed in detail below). On the other hand, the reduction in immunosuppression may not represent the proper answer for all infections, firstly since each drug has specific effects on some part of the immunological response, and stopping its usage may not improve the outcome; secondly, the interruption of immunosuppressive medications could cause an inflammatory relapse after recovery, with potential severe reactions (i.e., reconstitution syndrome after tuberculous meningitis) [5,6,7,8]: at least, for the risk of concomitant or subsequent rejection, additionally considering that some infections (e.g., BK virus) may also be contemporarily observed during rejection episodes. For all these reasons, immunosuppressive reduction requires careful evaluation, also taking into account every other factor that may cause an aggravation of the disease state and could be safely and more easily corrected (e.g., neutropenia with G-CSF or IgG deficit with endovenous immunoglobulins).

Of note, in this setting, preventive measures are tailored according to the predictable risk of infection [1], which, as expressed above, varies after time but is also strictly dependent on specific donors’(e.g., serological status for previous viral infection, cause of death, time in ICU) and recipients’ characteristics. After the transplant, prevention should include the acknowledgment of potential prophylaxis regimens for some conditions (i.e., CMV) and implementing commonly adopted strategies to reduce the risk and the impact of transmissible diseases (e.g., rapid removal of urinary/central venous catheters).

## 3. Immunosuppressants Role and Selective Immunosuppression Load

Transplanted patients were collectively considered immunosuppressed, and, intuitively, every infection may benefit from a reduction in immune impairment. However, as mentioned above, the picture is more complex since every disease has different characteristics, every drug has specific effects on the immune system, and every reduction in immunosuppression may increase the risk of rejection [6].

Analyzing the most common immunosuppressants in KTRs, Thymogloulines (ATG), now widely adopted in induction and T-cell-mediated acute rejection, are depletive agents that determine T- and B-lymphocytes rapid reduction. ATG were prevalently associated with latent virus reactivation and lysis syndrome, with a possible predisposition to bacteria infections [9,10]. All these risks might be amplified in the setting of acute rejection if ATG were preceded by steroid boluses or associated with increased maintenance immunosuppression [6].

Corticosteroids are widely used in induction and maintenance protocols, altering T-cell activation, proliferation, migration, and cellular response (negative modulation in PAMPs/DAMPs and multiple cytokine pathways and reduction in neutrophil adherence) [11,12]. Combined with reduced wound healing, these various effects determine an increased risk of bacterial infections and viral reactivation [1,6].

Mycophenolate mofetil primarily impairs T-cell function through reduced proliferation/apoptosis enhancement, alteration of cytokine receptor expression, and adhesion; its use is mainly related to chronic viral infections (i.e., CMV, BKV) [13,14].

Calcineurin inhibitors also profoundly affect T-cell activation and proliferation, with additional effects on CD4 T-cell differentiation, Treg expansion, and FOXP3 production [15,16,17,18,19]. These drugs also reduce neutrophil and macrophage bacterial phagocytosis and downregulate Toll-like receptor function [20,21]; however, their prominent role on the T-cell axis was confirmed by an increased risk of viral infections (e.g., CMV, *HSV*) [1,6].

mTOR inhibitors impair both T-cell and innate immune systems, such as altering the Th1 subset reducing IL-12 and IFN-gamma and negatively modulating the oxidative neutrophils burst; they are also directly related to surgical site infection (for their effect on wound healing) and have direct pulmonary toxicity [22,23,24,25]. Their potential antiviral effect is debatable, with possible advantages in some settings (CMV, BKV) [26].

The heterogeneity and increased number of monoclonal antibody/fusion protein now included in the therapeutic management of transplanted patients expanded the scenario of potential interactions and infection risk: for example, anti-CD20 (i.e., Rituximab), adopted in different settings for antibody-mediated damage or relapsing disease (i.e., membranous nephropathy), causes substantial B-cell depletion and hypogammaglobulinemia [27] and is directly related to HBV reactivation; Belatacept, a costimulatory inhibitor now adopted in cases of impaired renal function or CNI toxicity, logically altered T-helper activation of B cells and Treg expansion. EBV-Ig negative recipients should not receive this drug for the subsequent risk of EBV-related PTLD [28,29].

IL-6 inhibitors, the most promising drugs for chronic antibody-mediated rejection, altered neutrophil count survival, oxidative burst, and phagocytosis [30,31]; some case series suggest a potential risk for intestinal perforation after their use in KTRs with a significant history of diverticulitis [32]. Use of terminal anti-complement drugs (i.e., Eculizumab) required prior vaccination or antibiotic prophylaxis to avoid meningococcal infection, considering the crucial role of membrane attack complex formation in controlling encapsulated bacteria [33,34].

An additional issue derived from the possible monitorization of drug-induced immune deficit; most tests have been proposed, including intracellular adenosine triphosphate levels (iATP) [35,36], interferon-gamma (IFN-γ) based assays [37], and composite scores including cells count and molecular analysis [38,39]. A predictive role of Torque teno virus (TTV), an anellovirus with no pathogenic role besides active replication according to the immune “state”, has also been investigated [40,41]. However, all these methods have different specificities in potential risk prediction but were not explicitly tested in protocol with drug titration and, at least, failed to document a clear drug-infection relationship [6] directly.

**Figure 1:**
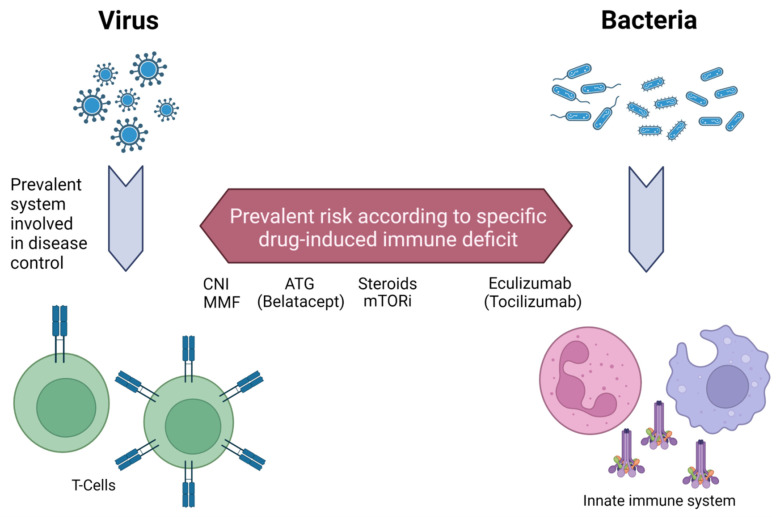
**A schematization of immune system prevalent activation during viral and bacterial infection and different effects of common immunosuppressants**. T-cell activation is crucial in viral infection control, whereas the innate immune system prevalently mediates response against bacteria. Immunosuppressive drugs have multiple effects on the immune system, with prevalent inhibition of T-cells (i.e., CNI) or innate system (i.e., Eculizumab), and consequent careful reduction during infective episodes should take into account their different profile. CNI: calcineurin inhibitors; MMF: mycophenolate mofetil; ATG: anti-thymocyte globulin; mTORi: mammalian target of rapamycin inhibitors. This figure was created with BioRender.com.

A graphical scheme of the overall drugs’ effects on the immune system and mechanism involved in infection response is reported in Figure 1.

Despite the general considerations that T-cell function should be enhanced in viral infections, whereas the innate immune system controls bacterial episodes, every disease has specific characteristics. Immunosuppressive modulation could enhance/modify the infection course, but disease severity and availability of effective antiviral/antibiotic therapies are also crucial parts of this picture.

A reduction in CNI could be considered in cases with persistent viral infection (e.g., CMV and BKV). CMV was related to all T-cell-depleting agents and high-dose steroids use [1,42,43,44]; BKV, which is additionally aggravated by the absence of specific antiviral drugs, often requires a step-by-step approach with CNI and MMF reduction to control the disease [45]. In these settings, mTOR may exert a “protective” role, even though this consideration is not univocally accepted [26,46]. However, no study specifically focused on immunosuppressive management among KTRs, and a tailored approach should be preferable considering disease severity and patient/graft outcomes.

## 4. Bacterial Infections

### 4.1. Urinary Tract Infections

Urinary tract infections (UTI) account for most of the infectious episodes in KTRs (from 45% to 75% of all infections in ≈25% of all transplanted patients) [47,48,49] and may cause severe sequelae, including sepsis, acute rejections, reduced graft function, and, at minimum, allograft lost, with increased mortality risk [48,50,51,52].

Apart from classical risk factors (female sex, advanced age, catheterization, diabetes mellitus), some specific conditions, including immunosuppressive therapy [53], increase the probability of UTI, such as the presence of a double-J ureteral stent and the eventual occurrence of delayed graft function [50].

Typically, UTI derives from ascending, gram-negative bacteria (up to 90 percent according to literature data), including *Escherichia coli*, *Enterobacter cloacae*, *Pseudomonas aeruginosa*, and *Klebsiella* spp. *(K. pneumoniae*, *K. oxytoca*) [48,51,52]. Among Gram-positive bacteria, *Enterococcus* spp. are more frequently observed [47,51,54]. However, other organisms (*Staphylococcus* spp., *Streptococcus* spp., *Corynebacterium urealyticum)* are rarely documented but could be significant in persistent catheterization or concomitant surgical site infection [52,55,56].

Although available guidelines do not express clear indications [57], screening for asymptomatic UTI may be reasonable in the first one to two months of KT (for example, at two, four, and eight weeks) [58], also considering that patients with untreated UTIs in this period could experience an increased rejection risk and adverse outcomes [50,52,59]. Similarly, some authors proposed different antibiotic (trimethoprim/sulfamethoxazole (TMP), fosfomycin) and nonantibiotic (vaginal estrogen, cranberry products, Methenamine Hippurate, L-methionine, probiotic prophylaxis regimens) treatments, despite all these approaches failing to demonstrate a clear benefit, being, for drug approaches, per contrast, potentially associated with an increased risk of antibiotic resistance [49,57,58,60,61,62].

Clinical symptoms include classical cystitis or, in case of complicated UTI, constitutional alteration (fever, nausea, vomiting, malaise, fatigue) with tension or pain in the graft site [50,51], in spite of the fact that, as for other infections, KTRs may experience no urinary/slight symptoms [57]. Pyelonephritis could be observed more frequently than in the general population, and despite being considered a relative “favorable” complication in past years, it is now recognized as a possible detrimental factor for allograft function [63].

Diagnosis should be made with urinalysis, blood tests for acute infection (e.g., WBC count, C-reactive protein), and, ideally, a radiological confirmation for pyelonephritis exclusion in case of constitutional symptoms or recurrent episodes [49,52]. Therapy should be tailored according to microbiological analysis, patients’ characteristics and risk factors, and microbial situation of the geographical area, and ideally, of the specific transplant ward [52,57]. Anatomical conditions favoring infection (e.g., urinoma, fistulas, lithiasis) should be corrected, especially after recurrent or complicated UTI [49,57,64].

In uncomplicated UTI, empiric regimens (Table 2) include fluoroquinolones (ciprofloxacin, levofloxacin), cephalosporins, and adding amoxicillin or nitrofurantoin in case of suspected Enterococcal etiology. Duration varies according to center protocol but should be limited to five to seven days to reduce the risk of resistant strains [52,57,65]. In complicated UTIs, intravenous antibiotics should be adopted, and an empiric regimen (i.e., IV cephalosporins, piperacillin-tazobactam, or meropenem) should be rapidly modified after microbiological assessment, if available. Treatment must be prolonged in the case of pyelonephritis and at least up to 14–21 days [49,52,57].

### 4.2. Respiratory Infections

Solid-organ transplanted patients, including KTRs, are at multiple risks of bacterial respiratory infections due to the direct (i.e., pulmonary toxicity of mTOR inhibitor drugs) [66] and indirect (i.e., neutropenia) reduction in lung defense mechanisms caused by immunosuppressive agents [67,68]. As for other infections, the period after transplantation suggests a different etiology: reactivation of a previous infection, donor transmission, or hospital-acquired Gram-bacilli during the first month, and opportunistic agents and intracellular bacteria (e.g., *Mycoplasma pneumoniae*) between one and twelve months after transplant [1,67]. Note that viral infection may represent the first hit in lung damage with consequent superinfection by different bacteria, including Nocardia species [1,69].

Management of respiratory infection needs a prompt and multidisciplinary approach to obtain a specific diagnosis. First of all, radiological analysis is required, and in this context, thoracic CT scan is a cornerstone of the diagnostic workup for its superior ability in disease identification and follow-up [1,67]. Apart from classical bacterial presentation (lobar, focal, or multifocal consolidations), some different pictures may suggest different pathogenesis (peribronchiolar opacity or bronchopneumonia for atypical agents including *Mycoplasma* spp., atypical Mycobacteria, *Chlamydia*, *Neisseria*, *Haemophilus* spp.; nodular infiltrates for *Legionella* spp.; subacute disease with peribronchovascular or miliary abnormalities in *nontuberculous mycobacteria*; cavitation in *Nocardia* spp.) [1].

As for all the infections in transplant settings, specific microbiological isolation is highly desirable (especially in severe/relapsing disease) and could be obtained through bronco-alveolar lavage or, eventually, lung biopsy in patients with inconsistent BAL results/differential diagnosis with suspicious proliferative illness [1,67].

Community-acquired bacteria causing respiratory tract infection include *Streptococcus pneumoniae*, *Haemophilus influenzae*, *Mycoplasma* spp., *Legionella* spp., and *Chlamydia* spp.; *Pseudomonas* spp., enteric Gram-negatives, and *Stenotrophomonas* spp. could be observed in nosocomially-acquired cases or during the early post-transplant course [67,68,70,71].

Empiric therapeutical management (Table 2) should consider patient characteristics, clinical conditions, and microbiologic subsetting (including environmental exposition) [1,67]. In stable outpatients, an initial approach with beta-lactam agents or fluoroquinolones is usually suggested; the eventual addition of anti-methicillin-resistant *Staphylococcus aureus* (MRSA) or *Pseudomonas* spp. drugs could be considered based on the patient’s history and clinical setting. In hospitalized recipients a beta-lactam (±coverage on MRSA and *Pseudomonas* spp.) and an additional drug with a direct effect on intracellular pathogens (*Mycoplasma* and *Legionella* spp. in adults) are highly recommended [67].

Other bacterial infections with possible respiratory involvement (*Nocardia*, Tuberculous/nontuberculous *mycobacteria*) are discussed separately.

### 4.3. Mycobacteria

*Mycobacterium tuberculosis* represents a significant healthcare problem, with a worldwide death toll of about one million people per year [72]. However, the disease distribution directly correlates with economic status, with endemic diffusion in low-income areas [72,73]. Most patients develop a latent infection, classified by WHO as a state of persistent immune response to stimulation by Mycobacterium tuberculosis antigens with no evidence of clinically manifested active TB disease [74], which may become active in case of reduced host defenses (i.e., for transplant immunosuppression) [73,75,76]. Not surprisingly, solid organ transplanted patients are an “at-risk” population with high incidence and adverse outcomes, including allograft impairment [73,77,78,79]. Clinical tuberculosis after transplant mainly occurs after reactivation of latent disease (potentially but rarely donor-derived), but also as a “primary” disease due to increased susceptibility; in this case, the prognosis could be severe, with potential miliary involvement [79,80,81,82]. Additionally, as for other infections, classical symptoms (fever, cough) may be absent with silent presentation [73,79].

Based on these assumptions, patients should be routinely screened for latent infection before transplant, especially those born or who have lived in endemic areas. WHO considered three tests for screening: Tuberculin skin test (TST), QuantiFERON1-TB (QFT), and Gold In-Tube and T-SPOT1 T [1,73,74,79]. The last two, both evaluating interferon-gamma release, are now increasingly important because they are more sensible, specific, and, opposed to TST, have no limitations in patients with chronic kidney disease or on immunosuppression [83,84,85]. Despite these advantages, the non-uniform availability, combined with high costs, limited their use, especially in endemic areas [73,79].

Patients with latent infection should ideally receive a prophylaxis regimen before kidney transplant or, if transplantation occurs during this time, stop the drug(s) for the early period after transplant and then restart to complete the schedule. According to available guidelines, a prolonged course with isoniazid is preferable, with a strict follow-up for monitoring adverse events which could be relatively common with this drug [73,79,86]. Isoniazid is also the cornerstone of active disease treatment, although the association with other drugs (e.g., rifampin) poses significant problems for the risk of hepatotoxicity and drug interference on hepatic metabolism, with a noticeable impact on immunosuppressive medications [73,79,87,88]. Standard prophylaxis and therapeutic approaches are summarized in Table 2.

Recently, nontuberculous Mycobacteria are acquiring significant importance, with not less than 25 species able to cause disease in transplanted patients (including the most common *Mycobacterium abscessus* and *Mycobacterium avium complex*) [89]. All nontuberculous mycobacteriosis significantly impact morbidity and mortality with frequent dissemination, difficult isolation, considering their ubiquitous diffusion, and problematic treatment requiring prolonged therapy with direct toxicity, risk of side effects, and multiple interactions with different drugs [90].

### 4.4. Nocardia Species

*Nocardia* spp. are gram-positive worldwide-distributed bacteria included in the Actinomycetales order [91]. Clinical infection develops only in an immunocompromised host, identifying nocardiosis as an opportunistic disease [92].

*Nocardia asteroids* characterized most of the nocardiosis diagnosed in KTRs [93]. However, many other species could be observed in the transplanted population, also with different clinical courses (i.e., *N. farcinica*, typically associated with severe central nervous system (CNS) involvement) [94]. Incidence in the transplanted population, generally above 3%, dramatically varies according to the geographical area, type of transplant (increased risk in lung recipients), and net state of immunosuppression, with increased risk in patients with a history of recent CMV infection, treatment with depletive agents/steroids for induction or rejection, and tacrolimus use at high doses [92].

The lung is the primary site of infection, followed by cutis/soft tissues and CNS, despite all organs being involved [92,93]. Single or even multiple abscessualization is the typical presentation of CNS nocardiosis. However, it may occur with slight or absent neurological symptoms, justifying the adoption of rapid neuro-radiological imaging (CT/MRI, according to local protocols) in every patient with documented *Nocardia* spp. isolation on cultures [92,93]. 

*Nocardia* spp. isolation is troublesome and costly, despite the necessity of its culturing for the evaluation of antibiotic resistance [92,93]; based on this assumption, the microbiological unit should be alerted and consulted in every case of suspicious nocardiosis. Some authors also proposed PCR techniques for rapid isolation [92]. High-dose sulfonamide agents are the cornerstone of therapy, with prolonged treatment (three to six months) usually followed by a subsequent time of “prophylaxis” to prevent relapse of disease, commonly observed in transplanted patients (as summarized in Table 2). Other drugs could be included in the first-line regimen in case of severe clinical course or after documentation of *Nocardia* spp. resistance on culture and/or insufficient clinical response [92,93]. The prognosis is generally severe with high mortality, especially in CNS involvement [92].

### 4.5. Listeria Monocytogenes

*Listeria monocytogenes* is a gram-positive, aerobic, and intracellular bacteria that, in the general population, causes gastroenteritis after ingestion of contaminated food. Despite rare frequency [95], in an immunocompromised host, including transplanted patients, Listeria may spread with bloodstream and CNS involvement, with high mortality [95,96,97]. Diagnosis should be performed with blood culture or PCR on CSF fluid in case of neurological impairment; first-line therapy (Table 2) includes high-dose ampicillin or amoxicillin. In allergic/intolerant patients, trimethoprim-sulphamethoxazole is considered a feasible approach [98,99]. Aminoglycosides have been proposed as alternative or synergistic drugs, however, apart from their intrinsic nephrotoxicity, fail to cross the blood barrier, with consequent obvious limitation in patients with meningitis [98]. Note that Listeria is intrinsically resistant to cephalosporins and shows reduced susceptibility to fluoroquinolones [100].

## 5. Viral Infections

### 5.1. Cytomegalovirus

*Cytomegalovirus (CMV)*, a herpes-virus family member, may cause significant infection in solid organ transplanted patients, including KTRs [1,43]. *CMV* infects most of the general population during infancy but generally remains at a latent state without symptoms, becoming significant only in subjects with persistent/transient immunosuppression or during pregnancy for the potential risk of fetal malformation [1,101].

In the transplant field, as for many other pathogens, especially viruses, *CMV* infection appears as crucially determined by the host response to many factors, primarily to the immunosuppressive load [1,43,44]. Not surprisingly, the *CMV* infections commonly occur in the first period after transplant (<six months) but could be transiently noted at every time during the transplant course after an increase in immunosuppressive drugs/impairment of the immune response (e.g., treatment of acute rejection, severe infection, an unintentional increase in drug levels for intestinal problems). The most severe infections occur in patients with negative serology before transplant (R−) who received a graft from a CMV-Ig positive donor (D+). With no prevention, almost every D+/R− develops *CMV* viremia (which, in these cases, constitutes a “primary infection”), and half of them experience clinical symptoms. R− patients are an essential at-risk group that may also develop infection after transfusion and sexual activity with CMV-Ig positive partners [1,43,44,46].

Additionally, clinical symptoms in transplanted patients are usually mild or absent. However, the symptomatic picture occurs more often in this subgroup, including fever, leukopenia, muscular tenderness with myalgia, increased liver enzymes (AST, ALT), and gastrointestinal involvement (gastritis/colitis with different extensions/or mucosal ulceration). As for other Herpesviridae, the neurological involvement with encephalitis and associated retinitis is described but uncommon [1,43,44,46].

A diagnostic workup includes evaluation of viremia with quantitative molecular assays (NAT) and histological assessment (i.e., CMV inclusion) in the case with disseminating disease and severe organ damage (e.g., gastritis/pancolitis); serological evaluation discriminates between pre-transplant “seropositive” patients but is not helpful for diagnosis or disease monitorization [46,102,103]. Response to treatment requires at least one week, considering the standard time after the first evaluation [46,103,104].

Different approaches for disease prevention are proposed in the literature, including general prophylaxis or pre-emptive therapy in at-risk patients (i.e., D+/R−, induction protocol with depletive antibodies) [46,103]. Both strategies have pros/cons, but to date, most centers, including ours, prefer a tailored approach with pre-emptive therapy and strict viremia monitorization. In any case, a residual percentage of patients, especially in the D+/R− group, experienced *CMV* viremia after prophylaxis [105]. We adopted the “standard” pre-emptive regimen of six months in our center with oral valganciclovir [46,103]. No consensus is still available for viremia threshold without symptoms: most centers consider a persistent viremia > 1500–2000IU/mL to start treatment [46,106].

In documented *CMV* disease or significant viremia, the treatment included intravenous ganciclovir or oral valganciclovir (reserved for patients with mild illness and no gastrointestinal involvement). Therapy could be stopped after two negative tests. Patients with primary or severe disease may receive prolonged treatment after negativization. In the case of concomitant hypogammaglobulinemia, adjunctive therapy with CMV-Ig could be considered [46,103].

In patients with no remission or persistent viremia, the emergence of resistant strains should be tested; ganciclovir resistance is more common in patients with severe disease or inadequate therapy [107]. In these cases, the second-line treatments include foscarnet and cidofovir without response to high ganciclovir doses [1,107]. Several alternative agents are under evaluation (e.g., brancidofovir); recently, results of letermovir prophylaxis have been published, showing a similar rate of *CMV* breakthrough but significant interaction with tacrolimus dosage [108]. Common side effects included neutropenia for ganciclovir and valganciclovir and direct renal toxicity for cidofovir and foscarnet(which is additionally associated with salt-wasting) [1,107]. Most adopted therapeutic managements and prophylaxis are summarized in Table 2.

### 5.2. Polyomaviruses

The name of this family is derived from the first discovery of SV40 in a cell-line derived from Africa Green Monkeys adopted for poliomyelitis vaccine production. The two members with importance in infectious transplant disease are *BK* and *JC*. Both have ubiquitous distribution in the general population without relevant symptoms, but may cause severe disease in immunocompromised subjects [109,110,111].

In detail, *BK* is associated with *BK* nephropathy, ureteral stenosis, or hemorrhagic cystitis; *JC* rarely causes direct viral nephropathy but is associated with progressive multifocal encephalopathy. According to literature data, in kidney transplantation, the incidence of *BK* infection varies from 1 to 10% [110]. The infection is generally derived from donors; renal tubular cells maintain the virus in a latent state that reactivates after immunosuppression. Not surprisingly, all conditions associated with reducing immune defenses (old age, depletive induction, treatment for acute rejection) are associated with an increased risk of *BK virus-associated nephropathy* (BK-VAN); a previous transplant lost for BK-VAN is another crucial risk factor for BK-VAN [110,111].

The BK virus was suspected in past decades after verifying positive viral inclusions in urinary cells (decoy cells). To date, it is well known that BK viruria may occur without symptoms or development of BK-VAN, and monitorization is performed with BK-DNA at specific time points (usually monthly for nine months, then every three months for up to two years after transplant); diagnosis could be suspected in case of concurrent clinical symptoms (acute renal impairment without hydronephrosis, evidence of ureteral stenosis without documented urological causes) with viremia but should be histologically confirmed [109,110]. In the case of BK-VAN, a kidney biopsy is also required for grading according to BANFF classification; of note, BK virus may cause a tubulitis that could be relatively indistinguishable from that caused by cellular mediated rejection without immunohistochemical staining with anti-SV-40 Ab (able to identify all polyomaviruses) [112,113]. Additionally, inappropriate steroid boluses are a documented cause of BK-VAN progression [109].

*JC* may rarely cause polyomavirus nephritis with similar features of *BK*, positive anti-SV40 antibodies staining on kidney biopsy, but negative BK-DNA. *JC* investigation may be run out after evidence of CNS symptoms of unknown origin (progressive alteration of mental status, seizures) [111].

Treatment of polyomavirus infection (Table 2) is a matter of debate. As also performed in our center, many authors considered a step-by-step approach with an initial reduction in immunosuppression (firstly anti-metabolite and calcineurin inhibitors) according to the severity of the disease and the patient’s characteristics [109,110]. Additionally, IVIg has been widely used based on small but positive observational data [114,115]. Although this effect has recently been partially questioned, some studies suggested mTOR inhibitors’ protective role [116,117]. Other antiviral therapies could be considered in cases of severe BK-VAN with persistent viremia. Although no specific treatment is available, some drugs demonstrated an antiviral effect. Cidofovir and leflunomide have been more intensively investigated, even though adverse events are relatively common during therapy (acute tubular necrosis for cidofovir, leukopenia for leflunomide) [109,110]. Generally, a more intensive approach with rapid reduction inimmunosuppressive therapy and addition of antiviral drugs is required in case of *JC* involvement, especially with severe CNS symptoms [111].

Unfortunately, BK-VAN causes graft failure in a significant percentage of patients [110]. Retransplant in these cases typically requests a negativization of BK-DNA for at least six months; in case of persistent viral load, a transplantectomy should be considered, despite not abolishing the risk of BK-VAN recurrence in the new graft [109,110].

### 5.3. Epstein-Barr Virus

*Epstein-Barr Virus* (EBV), a member of gamma Herpesviridae, commonly infects young children worldwide, and almost every adult subject develops antibodies during their lifetime [1]. In the general population, *EBV* infection may occur without symptoms or be associated with fever and respiratory symptoms (often in children) or, especially in young adults, as infectious mononucleosis with fatigue, fever, and asthenia associated with lymphadenopathy, hepatosplenomegaly, and, in some cases, hepatitis [118].

KTRs with negative Ig anti-EBV may develop the primary disease, and this condition is also exposed to a high risk of post-transplant lymphoproliferative disease (PTLD) [119,120]. As for other viruses, the infection is more common in the first year after transplant; the clinical course may be silent or with diffuse symptoms (including, additional to classical presentation in the immunocompetent host, B cell lymphocytosis, meningitis, and pancreatitis). Patients with incomplete viral clearance or persistence viremia should be monitored for the high risk of PTLD [119,120].

To date, no indication for pre-emptive therapy is available, in D+/R−, as well; however, close monitoring with a periodic determination of EBV-DNA is warranted [119,120,121]. Treatment is generally tailored according to the patient and the disease severity, firstly considering a cautious reduction in immunosuppression, which is most effective ([119,120,122] and Table 2). Ganciclovir could be considered in patients with persistent viremia, despite its unclear role in preventing PTLD [119,120].

### 5.4. Other Common Herpes Viruses (Herpes Simplex and Varicella Zoster Viruses)

Herpes simplex infection is caused by *Herpes Simplex Virus type-1* (*HSV*-1) and 2 (*HSV*-2), and is characterized, in the general population, with oro-labial and genital lesions in *HSV*-1 and *HSV*-2, respectively (1, 2), and a relatively high incidence (almost 50% with positive antibodies against *HSV*-1 in 40–49-year-old subjects) [123,124].

After primary infection, the virus remains latent in peripheric nervous sensorial ganglia, with potential reactivation during a period of reduced host responses [125]; therefore, not surprisingly, KTRs experienced prolonged viral shedding and more severe clinical symptoms and reduced response to available treatments [123,126,127]. Despite donor transmission being described, it rarely caused *HSV* infections, which are commonly dependent on viral reactivation, especially after anti-rejection therapies. Clinical presentation includes classic mucocutaneous lesions and disseminated disease with esophagitis, hepatitis, pneumonitis, CNS involvement with meningoencephalitis, transverse myelitis, recurrent lymphocytic meningitis, or keratitis [123].

Apart from classical symptoms being routinely diagnosed without laboratory testing, PCR is required, especially in case of differential diagnosis and unusual or severe presentation (i.e., on CSF in patients with CNS symptoms) [123].

Treatment (Table 2) included a short course (usually five to seven days) with oral nucleoside analogs (acyclovir, valacyclovir, or famciclovir [128]) in cases with the limited mucocutaneous disease. According to clinical response, patients with diffuse lesions, organ involvement, or CNS infection should be treated with intravenous acyclovir for 14–21 days [123,129]. Low improvement after prolonged therapy or relapsing disease should be screened for acyclovir resistance, reaching 10% in some case series [129].

Similar to *HSV*, *Varicella-Zoster virus (VZV)* may rarely occur as a primary disease but, in these cases, could be associated with pneumonia or multi-organic involvement. Reactivation with limited dermatomal lesions remains the typical presentation, although transplanted patients may experience disseminated disease or CNS/visceral manifestations, also without skin lesions [130,131,132]. As for *HSV*, clinical diagnosis should be followed by PCR test in patients with severe disease, CNS symptoms, or atypical clinical course. Therapy includes acyclovir with intravenous administration in patients with severe disease (Table 2). Based on these assumptions, vaccination before transplant in the seronegative recipient is highly recommended [131]. Universal prophylaxis in seronegative transplanted patients (or no responders for *VZV* vaccination) has not been routinely suggested, additionally considering that most patients have combined negative *CMV* antibodies and already receive a nucleoside analog [131].

### 5.5. Therapeutic Advances: HBV, HCV, and HIV

Hepatitis B and C, and HIV, have constituted a barrier to kidney transplantation for many years, with the additional warning of potential donor transmission. Despite the incidence and specific treatments of these conditions being beyond the scope of this review, to date, both HBV and HIV infection may be managed with effective antiviral therapies, making KT safely allowed in patients with “controlled” chronic disease [133,134]; the picture is now most impressive for HCV, where cytosolic cell cycle and highly-effective drugs have ensured complete viral clearance in almost treated patients with very low adverse events and the opportunity to consider organ donation from HCV+ to HCV− recipients [135]. At the same time, the adoption of routinary PCR tests in donors with potential infection risk has dramatically reduced the risk of transmission [135].

### 5.6. Emergent Issue: West-Nile Virus

*West-Nile* is a Flavivirus transmitted by mosquitos; despite most infections in the general population being asymptomatic [136], in an immunocompromised host, *West-Nile* may cause CNS involvement with a severe clinical course [137,138]. Considering its kidney tropism, the donors could transmit the infection, posing the question of routine screening in endemic areas, which are rapidly spreading worldwide (now, the first cause of viral encephalitis in the USA) [139].

PCR testing for *West-Nile* on CSF should be included in every case of suspicious viral CNS infections [139,140]. To date, no specific therapy has been approved for *West-Nile* treatment; some authors suggest, next to the reduction in immunosuppression, the adoption of intravenous immunoglobulins ([139,140] and Table 2).

## 6. Sepsis in Kidney Transplanted Patients

KTRs are a population at high risk for bloodstream diffusion and sepsis [141] (ex 40% higher rate of sepsis than the general population [142]), which also represent a significant morbidity and mortality cause [143].

Additionally, to date, multi-drug–resistant bacteria (MDR) determine a significant health care problem, and, among them, the so-called ESCAPE pathogens (vancomycin-resistant *Enterococci*, methicillin-resistant *Staphylococcus aureus*, *C. difficile*, carbapenem-resistant *Acinetobacter baumannii*, carbapenem-resistant *Pseudomonas aeruginosa*, MDR, and carbapenemase-producing *Enterobacterales*) could be more frequently observed in KTRs for their intrinsic frailty on UTI infections (preferentially site of most of these MDRs) combined with long-hospital stay and time on ICU (both frequently longer in KTRs than hospitalized population) [144,145,146,147,148].

Despite bacteria and, among them, Gram-negative agents with urinary tropism causing most KTRs bloodstream infections [144], viral episodes (e.g., CMV) may cause a negative “addition” to the immunosuppressive state or directly be superimposed with bacteria diffusion, evolving into septic shock [1]. Other conditions (old age, diabetes mellitus, pneumonia as site of infection, underweight/obesity, alloreactive status) also increase the risk of sepsis or directly contribute to adverse outcomes [149].

Rapid detection of the specific pathogen, intensive management, and avoidance of common potential risk factors (i.e., prolonged use of central venous or urinary catheters) are evident in the septic management of KTRs. In this setting, adequate fluid management represents a significant challenge, considering that KTRs are at high risk for AKI and fluid overload [150,151].

No guidelines clearly stated how immunosuppressive agents should be managed during sepsis; intuitively, considering that most episodes are determined by severe bacteria dissemination, the goal is to increase innate immune response with associated avoidance of adrenal insufficiency (e.g., with hydrocortisone) [152]. In this way, a reduction/temporary withdrawal of CNIs/MMF seems to improve the infection response with limited rejection risk [151,153]. Once again, timing and rapid detection of the causal agent are pivotal elements, and further studies are needed to assess and standardize the correct approach in these situations.

**Table 2:** Most adopted approaches in common bacterial and viral infections according to recent literature data.

Bacterial Infections	Viral Infections
**Urinary tract infection (UTI)** [65,154]	**Citomegalovirus (CMV)** [46,103]
Uncomplicated UTI: 5–7 days of ciprofloxacin 250 mg orally twice daily/levofloxacin 500 mg orally once daily or oral cephalosporins (e.g., cefuroxime 250 mg twice daily); consider the addition of amoxicillin 500 mg orally three times daily or nitrofurantoin 100 mg orally twice daily in case of suspected *Enterococcus* spp. presence Complicated: piperacillin-tazobactam 4.5 g IV every six hours or meropenem 1 g IV every eight hours for 14–21 days	Prophylaxis: 6 months oral valganciclovir 900 mg twice daily Therapy: intravenous ganciclovir (5 mg/kg twice daily); consider oral valganciclovir (900 mg twice daily) in patients with mild disease without gastrointestinal involvement. Therapy could be stopped after 2–3 weeks with one or two negative tests (depending on analytic sensibility and disease severity)
**Respiratory tract infection** (**pneumonia**) [67]	**Polyomavirus** (**BK**) [109]
Stable/outpatients: beta-lactam agents or fluoroquinolones; consider the addition of anti-*methicillin-resistant* *Staphylococcus aureus* (MRSA) or *Pseudomonas* spp. drugs according to patients’ history and clinical setting Unstable/hospitalized recipients: beta-lactam agents (+/− coverage on MRSA and *Pseudomonas* spp.) + drug against intracellular pathogens (*Mycoplasma* and *Legionella* spp.) Active TB: four-drug regimen of isoniazid, rifampin/rifabutin, pyrazinamide, and ethambutol for the first 2 months followed by isoniazid and rifampin alone for an additional 4 months High-dose TMP (15 mg/kg/day) or linezolid for 3–6 months	Cautious CNI reduction and MMF/MPA reduction/stop IVIg (Conversion to mTORi) Without response consider leflunomide (100 mg for 5 days orally, followed by a maintenance dose of 40 mg or adjusted according to plasma trough concentrations) and/or Cidofovir (0.25–1.0 mg/kg at 1–3 weekly) Cautious reduction in the net immunosuppression (Ganciclovir)
**Tubercolosis** [79,86,87]	**Epstein-Barr Virus** (**EBV**) [120]
Latent TB: 9 months isoniazid; 4 months rifampin; weekly isoniazid/rifapentine × 12 doses Active TB: four-drug regimen of isoniazid, rifampin/rifabutin, pyrazinamide, and ethambutol for the first 2 months followed by isoniazid and rifampin alone for an additional 4 months	Cautious reduction in the net immunosuppression (Ganciclovir)
**Nocardia** [92]	**Herpesviridae** (***HSV*-1,2** [120] **and VZV** [131])
High-dose TMP (15 mg/kg/day) or linezolid for 3–6 months High-dose ampicillin or amoxicillin (2000 mg IV every 4 h); in allergic/intolerant patients consider trimethoprim-sulphamethoxazole (3–5 mg/kg IV every 6 h)	Limited mucocutaneous (*HSV*-1 and -2): oral nucleoside analogs (acyclovir, valacyclovir, or famciclovir) for 5–7 days Severe disease: intravenous acyclovir for 14–21 days Reduction in the net immunosuppression (IVIg)
**Listeria** [98,99]	**West-Nile virus** [140]
High-dose ampicillin or amoxicillin (2000 mg IV every 4 h); in allergic/intolerant patients consider trimethoprim-sulphamethoxazole (3–5 mg/kg IV every 6 h)	Reduction in the net immunosuppression (IVIg)

## 7. Conclusions

Infections are a significant issue in kidney transplanted patients, with a noticeable impact on morbidity and mortality. The spectrum of possible diseases, thanks to worldwide traveling and environmental modifications, is rapidly changing, as dramatically demonstrated by the actual pandemic state from SARS-CoV2 [154,155]. At the same time, the adoption of new drugs such as monoclonal antibodies may cause a reappraisal of “old” diseases but with atypical presentations. Clinicians must be aware of these continuous modifications, constantly updating their preventive strategies, clinical management, and therapeutic protocol to the varied scenarios.

## Figures and Tables

**Table 1:** The black box of consideration for kidney transplant bacterial and viral infectious diseases.

Implement All Available Preventive Strategies According to the Predictable Risk of Infection
**Obtain precise radiological and microbiological assessment**
Collect fluid for microbiological identification, ideally before empiric treatment (i.e., sputum or bronchoalveolar lavage in case of upper/lung infection or urinalysis in UTI)
According to the infection site, consider drainage, biopsy, or histological analysis in case of negative/inconclusive first-line tests
**Consider the selective immunosuppressive load** (**see Section 3 and Figure 1**)
**Pay attention to the potential risk of rejection after reduction/suspension of immunosuppressive drugs**
Viral infection may impair the immune system with increased rejection risk (i.e., CMV)
Consider the risk of inflammatory relapse in case of immunosuppression reduction after disease recovery (e.g., reconstitution syndrome in neural tuberculosis)

UTI: Urinary tract infection; PTLD: post-transplant lymphoproliferative disorders.
